# Electric charge and salting in/out effects on glucagon’s dipole moments and polarizabilities using the GruPol database

**DOI:** 10.1107/S2052520625001088

**Published:** 2025-02-24

**Authors:** Raphael F. Ligorio, Rasmus H. M. Gehle, Leonardo H. R. Dos Santos, Anna Krawczuk

**Affiliations:** a Georg-August-Universität Göttingen, 37077 Göttingen, Germany; b Department of Chemistry, Federal University of Minas Gerais, Avenida Pres. Antônio Carlos 6627, Belo Horizonte, Minas Gerais, 31270-901, Brazil; Universidad de Oviedo, Spain

**Keywords:** quantum crystallography, proteins, distributed polarizability approach, dipole moments, solvation effects

## Abstract

This work demonstrates the use of the GruPol database to predict the functional group dipole moments and polarizabilities of glucagon in the presence of NaCl, simulating an electric charge distribution on the protein’s backbone. A new feature of the database allows for the inclusion of ions on the protein backbone, effectively simulating a protein salt and predicting the impact on electrical properties. Glucagon was selected as a proof-of-concept molecule due to its relatively small chain, which enabled benchmarking against quantum mechanical calculations. Firstly, we simulated 70 different ionic configurations, varying the number of Na^+^ and Cl^−^ ions from zero to four NaCl moieties. Additionally, we investigated the effects of solvation under two distinct conditions: one involving just the peptide and water, and the other also including NaCl at a concentration of approximately 4.2 mol L^−1^. Regarding the *ab initio* results, GruPol showed good accuracy, with an angular direction error of around 10° and a 15% difference in the magnitude of the dipole moments. However, the error in polarizability values was higher, most likely due to the lack of an augmented basis set in the *ab initio* quantum calculations (M06-HF/cc-pVDZ). The database entries were generated using the same functional along with the aug-cc-pVDZ basis set. In solution, a high ionic concentration lowered the overall dipole moment, while the main components of polarizability increased.

## Abstract 2

This work explores the prediction of glucagon’s dipole moments and polarizabilities using the GruPol database, incorporating ionic effects and solvation conditions. The results highlight the influence of high ionic concentrations on the protein’s electrical properties, demonstrating good agreement with quantum mechanical benchmarks.

## 1. Introduction

Understanding protein stability, reactivity and overall functionality is a central objective for many researchers, as these insights can elucidate the specific roles of proteins in medical conditions (Gebauer *et al.*, 2021 ▸; Gonzalez *et al.*, 2020 ▸; Hu *et al.*, 2022 ▸), inform the design of artificial catalysts (Li *et al.*, 2021 ▸) and even guide the engineering of novel proteins (Lovelock *et al.*, 2022 ▸). Many of a protein’s characteristics are intrinsically linked to its electrical and electrostatic properties, including dipole moment and polarizability (the latter referring to a molecule’s ability to produce a dipole moment in response to an externally applied electric field). These features are key determinants of intra- and intermolecular interaction energies, thus being crucial in predicting protein–ligand binding affinities (Goel *et al.*, 2020 ▸; Vascon *et al.*, 2020 ▸), solubility (Vascon *et al.*, 2020 ▸) and lattice energies (Ma *et al.*, 2023 ▸; Spackman, 2018 ▸).

Proteins are highly sensitive to their environment. A particular behaviour may only be detectable under specific conditions, such as a particular pH value, which can significantly change the charges, and hence electrical properties, on the protein’s backbone (Kim *et al.*, 2024 ▸; de Resende *et al.*, 2024 ▸). It has also been well established that altering the salt concentration in a protein solution can either enhance or reduce the protein’s solubility (Duan & Wang, 2024 ▸) (known as salting in or salting out, respectively), affect its activity (Martin del Campo *et al.*, 2023 ▸) or facilitate its crystallization process (Majeed *et al.*, 2003 ▸). These observations suggest that accurate assessment of a protein’s properties requires careful consideration of the experimental conditions. For instance, cell organelles are not found in pure aqueous solutions; they exist in the cytosol, an environment containing various substances including salts. Due to the vast number of proteins, which are not easy to crystallize, the wide range of experimental variables and the lack of accurate experimental techniques, it is impractical to measure all possible combinations. Therefore, the ability to predict these properties accurately through theoretical approaches is of significant value.

With the use of *ab initio* quantum theoretical methods, it is possible to calculate electrical properties and reach the desired accuracy. However, a challenge remains, as most proteins contain a large number of atoms, making *ab initio* methods generally computationally expensive and often difficult to converge. To overcome these limitations, a database approach is frequently employed. This method relies on the transferability of electron density or wavefunction among functional groups within similar chemical environments, which can then be used to calculate electrical properties. For smaller fragments, such as individual atoms or recurring functional groups, the electron density is calculated and stored in the database for future use. Large molecules can then be broken down into these fragments, allowing the molecular properties to be reconstructed using the database. Several databases employ this approach, each with distinct objectives. For example, the MATTS database (Kumar *et al.*, 2019 ▸) utilizes aspherical pseudo-atoms to estimate electrostatic potential maps of small relevant biomolecules. The ELMAM2 database (Domagala & Jelsch, 2008 ▸), with similar purposes, emphasizes resource efficiency for faster predictions. The Generalized Invariom Database (GID) (Dittrich *et al.*, 2013 ▸) applies the Hansen–Coppens multipole model to transfer the electron density of functional groups (building blocks) across similar systems. A recent study (Treger *et al.*, 2023 ▸) demonstrated the prediction of refractive indices for metal–organic frameworks through a building block approach, reconstructing the framework from its individual moieties.

**Figure 1:**
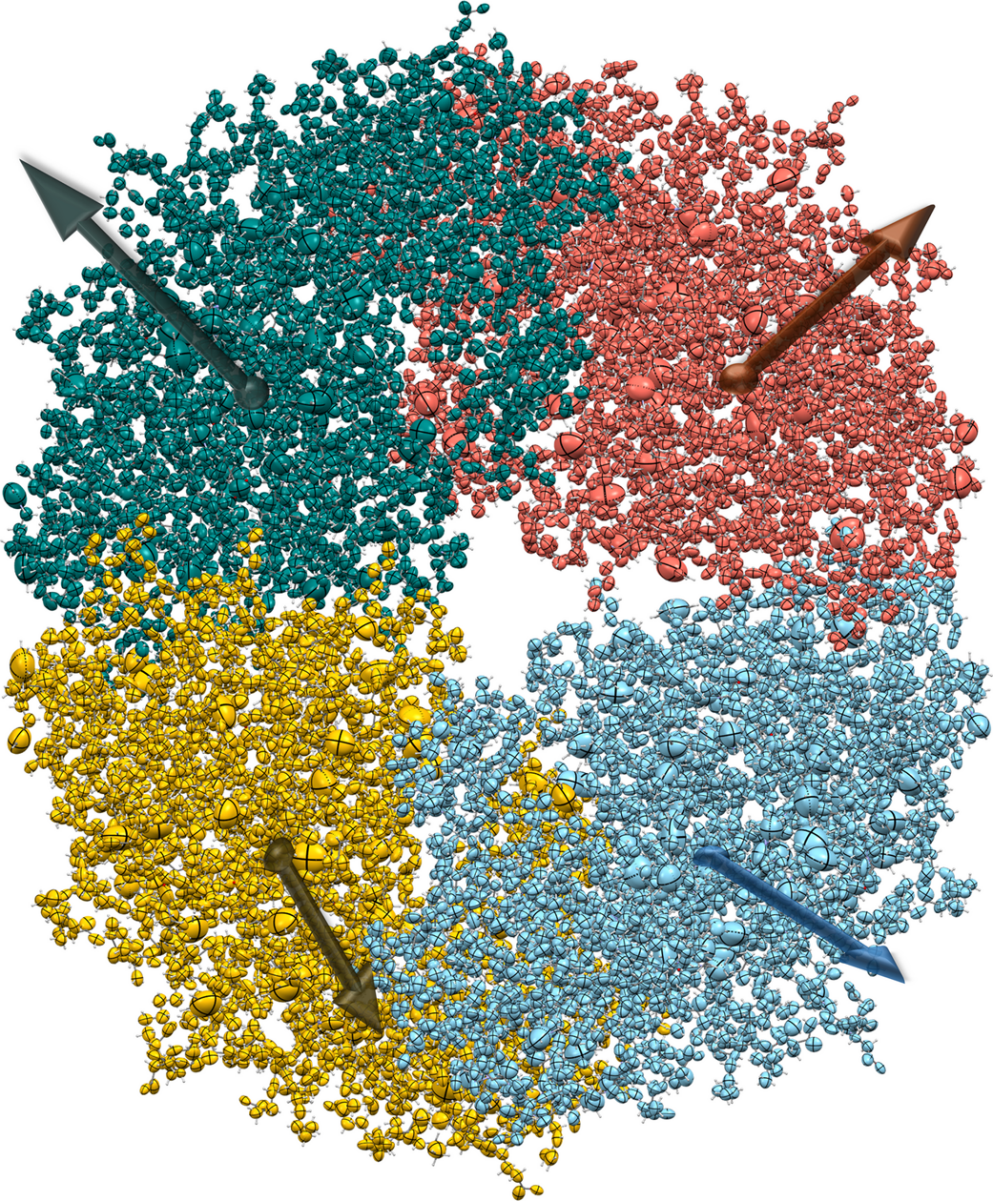
Building block polarizabilities and chain dipole moments for all four segments of glutamine amidotransferase (PDB refcode 1ao8). These properties are visualized with their original directions but with reduced magnitudes to fit the figure. Details and numerical values are provided in the supporting information.

Over the years, we have accumulated extensive experience in exporting electrical properties across similar systems in small molecules (Krawczuk *et al.*, 2014 ▸; Ligorio *et al.*, 2021 ▸; Dos Santos *et al.*, 2015 ▸; Dos Santos & Macchi, 2016 ▸; Jabluszewska *et al.*, 2020 ▸; Rodrigues *et al.*, 2023 ▸). Building on this expertise, a general database for polarizabilities was introduced by Ernst *et al.* (2019 ▸), based on the concept of atoms in molecules proposed by Bader (Laidig & Bader, 1990 ▸) and further refined by Keith (2007 ▸). This database concept has been expanded (Ligorio *et al.*, 2022 ▸; Ligorio *et al.*, 2023 ▸) and we recently launched GruPol, which focuses on the electrical properties of proteins (Ligorio *et al.*, 2024 ▸), consisting of the 20 most common amino acid residues. Our approach is not limited to polarizability; we have also incorporated group dipole moments and electrostatic potentials into the database. Additionally, GruPol employs a dipole interaction model (Applequist, 1977 ▸) to predict changes in these properties due to solvation, acknowledging the biological relevance of these interactions. GruPol focuses on predicting the properties of large molecules, as demonstrated in Fig. 1 ▸, where the apoprotein glutamine amidotransferase is used as an example due to its substantial size, consisting of four chains, nearly 2000 ▸ residues and approximately 30000 atoms. The molecule’s four chains are shown, each represented in a different colour. The dipole moment of each chain is illustrated with an arrow, while each building block is represented by its own polarizability tensor.

Due to the possibility of multiple protonation states in large proteins, resulting from the numerous ionizable residues, GruPol also incorporates a scheme to predict changes in dipole moments when simulations at different pH levels are required. Now, given the biological significance of neighbouring ions in the vicinity of the protein’s backbone, either altering the protein structure (Baldauf *et al.*, 2013 ▸) or changing its charge distribution (Lindman *et al.*, 2006 ▸), it seemed natural to extend our model to include their contribution to the electrical properties. Therefore, this paper focuses on benchmarking our latest approach to account for ions interacting with the protein and how they influence its dipole moment and polarizability. However, due to the size of glutamine amidotransferase, performing quantum calculations is impractical. For the purpose of guaranteeing good benchmarking, we opted for a smaller peptide, *i.e.* glucagon with 29 residues, as our test system. Its relatively small size allows for feasible *ab initio* quantum mechanical calculations, enabling us to validate the new GruPol approach. As a further step, we simulated the same peptide in two distinct aqueous environments, with and without the presence of ions, thus allowing us to evaluate the changes in glucagon’s electrical properties due to a solvated medium.

## 2. Methodology

We utilized the glucagon peptide to investigate the presence of ions and their influence on the electrostatic properties of proteins, such as dipole moments and dipole polarizabilities. This peptide comprises 29 amino acid residues, including eight primarily ionizable groups: three ASP residues, two ARG residues, one LYS residue, and the terminal groups CTER (–COO^−^) and NTER (–NH_3_^+^). Atomic coordinates of the peptide without additional ions were obtained from PDB entry 1gcn (Sasaki *et al.*, 1975 ▸) and kept frozen during the calculations. Missing H atoms were added using the *CHARMM-GUI* feature (Jo *et al.*, 2008 ▸). To maintain neutrality, Na^+^ and Cl^−^ ions were added in pairs along the protein’s backbone, coordinating to the ionizable residues, meaning that there is no excess of chlorine ions over sodium ions, or *vice versa*. All 70 possible configurations were taken into account, considering the complementarity between positive and negative charges: 16 with one pair, 36 with two pairs, 16 with three pairs and one with zero or four pairs. The properties of the ion-free compound were calculated assuming the terminal zwitterion state for which the database’s entries were created. Importantly, for all calculations, except the case without NaCl, all ionizable residues, when not coordinated to an ion, were considered charged, meaning that acidic residues (ASP and CTER) were deprotonated while basic ones (ARG, LYS and NTER) were protonated. Maintaining overall molecular neutrality is crucial as it eliminates the origin dependence of the dipole moment.

**Figure 2:**
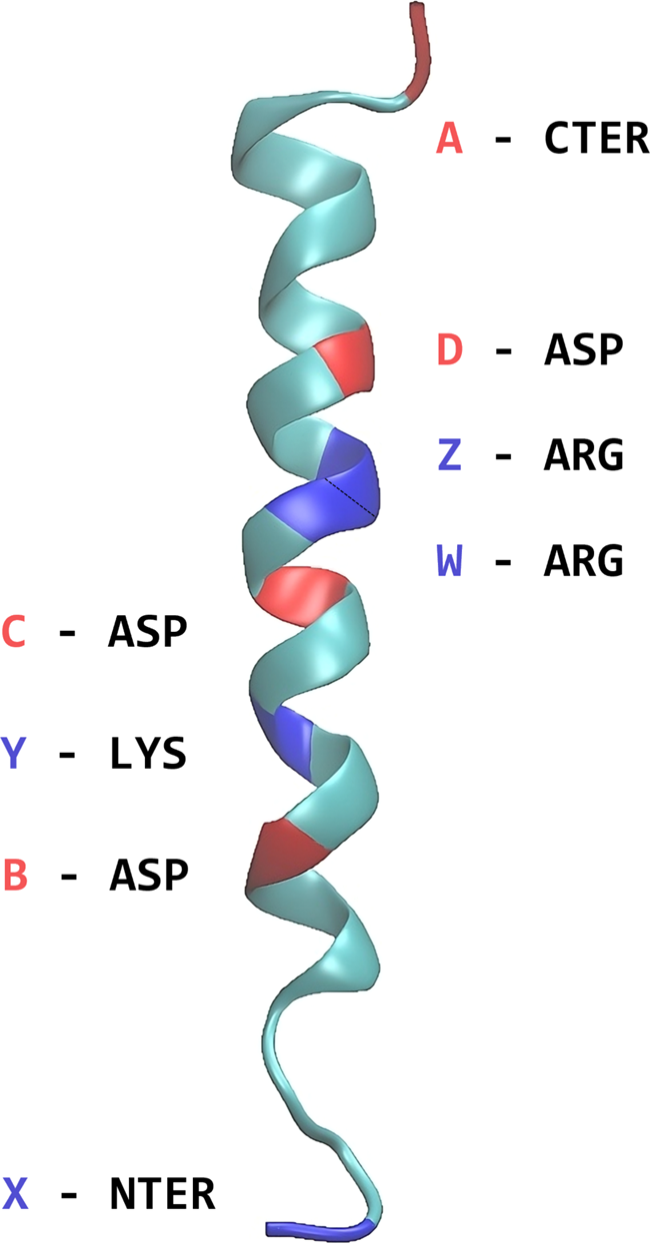
Representation of the glucagon molecule, highlighting the position of each ionizable residue. Negative residues are designated as A, B, C and D and are marked in red, while positive ones are labelled as X, Y, Z and W and indicated in blue.

We named the compounds containing up to three pairs of ions using a six-digit code: A, B, C and D for Na^+^ coordinated to the ASP and CTER residues, and X, Y, Z and W for Cl^−^ coordinated to LYS, ARG and NTER. Fig. 2 ▸ illustrates the positions of each ionizable group. For instance, if two pairs of ions were coordinated at residues A, C, Z and W, the code would be AC-ZW-. For the case without ions, we used the name ‘standard’, and when four ions were present the name was ABCDXYZW. Each compound was assigned a number: 1 for the standard, 2–17 for compounds with one NaCl pair, 18–53 for those with two pairs, 54–69 for those with three pairs and 70 for ABCDXYZW. The association of the symbols and numbering is provided in the supporting information.

*Ab initio* calculations were conducted to obtain molecular dipole moments and polarizabilities. The results were then benchmarked against the GruPol database (Ligorio *et al.*, 2024 ▸). This first step was undertaken to validate the new approach used by GruPol to account for the presence of the ions on the protein’s backbone. Additionally, to investigate the impact of solvation on the above-mentioned properties, glucagon was placed in a water box under two conditions: one without salt and the other with NaCl, where the ions were placed randomly. Further molecular dynamics simulations were performed to grasp the inherent flexibility of the macromolecule and its impact on the studied properties, as well as for properly coordinating the ions to the peptide, since they were initially randomly distributed in the water box.

### 2.1. *Ab initio* quantum calculations

*Ab initio* quantum calculations were performed using the *GAUSSIAN16* software (Frisch *et al.*, 2016 ▸) at the M06-HF/cc-pVDZ level of theory. This choice of the density functional theory (DFT) functional was based on our previous research, where we benchmarked DFT functionals against coupled-cluster singles or doubles (CCSD) calculations for the atomic and molecular polarizabilities of organic molecules (Ligorio *et al.*, 2020 ▸). The basis set selection was influenced by the size of the peptide. While the aug-cc-pVDZ basis set typically offers more reliable results due to its extended spatial coverage, thus increasing the polarizability by nearly 20% compared with the cc-pVDZ basis set, convergence issues with the large number of atoms in glucagon necessitated the use of the non-augmented version. Importantly, at least for smaller peptides, the differences in dipole moments between the two basis sets seem to be minimal (Ligorio *et al.*, 2024 ▸).

### 2.2. Including charges in GruPol predictions

The charge *q* of a specific ionizable residue was determined using the Henderson–Hasselbalch model, described by equation (1) for acidic groups and equation (2) for alkaline residues:
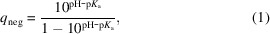

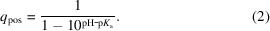
Charges are applied to the centre of ‘mass’ (here, masses are replaced by atomic numbers) of the individual ionizable building block, rather than to the centre of the entire residue The values of p*K*_a_ (Mellor *et al.*, 2011 ▸) used are given in Table 1 ▸.

It is important to note that the terminal groups show a charge with a counter-intuitive sign because they were previously classified as charged groups when the database entries were created. For example, one might expect the carboxylic CTER group to carry a negative charge. However, a positive charge is added because the database creation process has already accounted for the charged group. For these groups, we calculate the expected charge at a given pH and make adjustments based on an initial zwitterion model with a +1 charge for the NTER group and −1 for the CTER group. Thus, if the NTER group has an effective charge of +0.6 at a certain p*K*_a_, then instead of the original +1 we apply a compensating charge of −0.4 to achieve the intended charge dipole moment. Benchmarking against *ab initio* quantum calculations was performed by setting the pH to 7.3, which is the isoelectric point for glucagon as estimated by GruPol and which aligns well with the already reported literature value (Joshi *et al.*, 2000 ▸). At this pH, the charges assigned to ASP, LYS and ARG residues, and to the terminal groups, showed magnitudes close to 1. The other ionizable residues indicated in Table 1 ▸ presented minimum charges, apart from a single HIS residue with charge of approximately +0.3. It is important to note that, in the standard GruPol calculation, where no ions were included, charges are not added onto the protein backbone. This is equivalent to simulating the terminal zwitterion species. This was done to assess the intrinsic deviation of GruPol in estimating the properties of glucagon before applying the new salting in or salting out approach.

#### 2.2.1. Property corrections

The arrangement of charges along the protein backbone produces a dipole moment referred to as the charge dipole moment. The dipole moment arising from the polarization of electron density due to chemical bonding is known as the core dipole moment. The total dipole moment of the protein is the summation of these two components, the charge dipole moment and the core one,
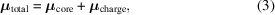
where **μ**_charge_ is given by
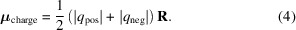
Here, **R** is a vector with its origin at the centre of the negative charges pointing to the centre of positive charges, given by
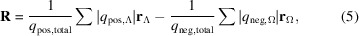
and **r** denotes the position of the centre of charge of each building block Λ or Ω that possesses a positive or negative charge, respectively.

The core dipole moment is adjusted by accounting for the electric field **F** originating in the presence of these charges, where *q* represents either a positive or a negative charge,
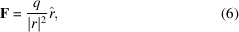
and |*r*| is the modulus of the vector connecting a given charge to a particular building block. 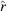 is a unitary vector with its origin at the charge, pointing to a given building block Ω. The total electric field experienced by a building block is the summation of each individual electric field **F**. The correction on the core dipole moment is then given by
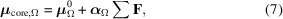
where **α**_Ω_ is the polarizability of a given building block Ω, and the superscript 0 indicates the initial dipole moment in the absence of any correction.

Note that, as reported in our previous studies (Jabluszewska *et al.*, 2020 ▸; Ligorio *et al.*, 2024 ▸), the presence of charges on the protein’s backbone does not significantly impact either the molecular polarizability or the corresponding values of its constituent building blocks, at least for small peptides. For this reason, the presence of charges on ionizable residues has no impact on GruPol’s polarizabilities.

#### 2.2.2. Presence of ions

To identify ions linked to ionizable residues, GruPol uses a distance threshold of 2.5 Å for chloride ions binding to LYS and ARG residues and the NTER group (H—Cl bond distance), and 3.5 Å for sodium ions associated with ASP, GLU and the CTER group (O—Na bond distance). When an ion is detected, the corresponding charged residue becomes neutral and no longer contributes to the charge dipole moment [equation (3)]. However, both the ionic charge and the residue charge continue to influence the core dipole moments of the other building blocks. In the second step, after correcting the core dipole moments due to the presence of charges on the protein’s backbone, a dipole interaction model (ADIM) (Applequist, 1977 ▸; Thole, 1981 ▸; Ligorio *et al.*, 2021 ▸) is employed to correct both polarizabilities and dipole moments due to the presence of the ions. The model assumes that the total electric field within each atomic basin is the result of both the external field and the field generated by the collective dipoles from neighbouring sites (Guillaume & Champagne, 2005 ▸; Mkadmh *et al.*, 2009 ▸),
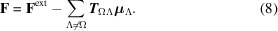

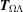
 represents the field tensor between the atomic basins Ω and Λ,
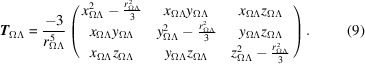
*x*_ΩΛ_ = (*x*_Ω_ − *x*_Λ_) represents the difference in the Cartesian *x* coordinate between the basins Ω and Λ, and *r*_ΩΛ_ denotes the corresponding interatomic distance. Using the total electric field and the original polarizability tensors, we can recompute the dipole moments, 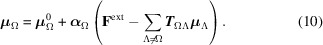


Finally, corrections on polarizabilities can be obtained by rearranging equation (10) in the form
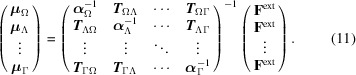
Summation of each row of equation (11) provides the corrected building block polarizabilities.

The close proximity of two polarizable sites can result in unrealistic values of dipole moments and polarizabilities. This occurrence is known as polarization catastrophe and was addressed by Thole (1981 ▸). To address this issue, a damping function is applied to the distance tensor, which here takes the form
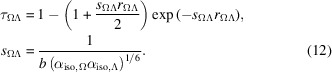
In this context, the function τ_ΩΛ_ scales the distance tensor 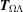 to decrease its magnitude at short distances. The coefficient *b* is an adjustable parameter chosen to match results from *ab initio* quantum calculations or experimental data, with a commonly used value of 2.6 (Lemkul *et al.*, 2016 ▸; Litman *et al.*, 2022 ▸), which was employed in this work as well. It is important to note that building blocks within the same molecule, such as the glucagon peptide, do not interact with each other, as these interactions were already accounted for during the development of the database itself. Therefore, changes in the dipole moment are exclusively attributed to environmental effects, which may or may not involve ionic solutions.

### 2.3. Molecular dynamics simulations

An initial cubic water box with a side length of approximately 84 Å was placed around the glucagon molecule under two different conditions. In the first setup, only the peptide and water molecules were included. In the second setup, NaCl units were also added, with an initial concentration of 3 mol L^−1^. After simulation, the final concentration of NaCl was found to be around 4.2 mol L^−1^.

The protein backbone was chosen in such a way to ensure its neutral terminal zwitterion state, as well as charged ASP, LYS and ARG residues, thus simulating the isoelectric point. An equilibration step was performed in the NVT ensemble, comprising 125000 steps with a time interval of 1 fs. Temperature control was achieved using the Nosé–Hoover (Hoover, 1985 ▸) thermostat set to 303.15 K. Following this, molecular dynamics simulations were performed in the NPT ensemble, controlling both temperature and pressure at 303.15 K and 1 atm, respectively (Berendsen *et al.*, 1984 ▸). The simulation was carried out for a total of 200000 molecular frames with a time interval of 2 fs, corresponding to an overall simulation time of 400 ps. Geometries were extracted every 2 ps. The first 100 conformers were discarded to ensure proper volume accommodation after the equilibration steps. The 100 final geometries were used as input for GruPol database. All molecular dynamics simulations were performed utilizing the *CHARMM* additive force field (Version 46b1; Brooks *et al.*, 2009 ▸), whereas input files and water box placement were done employing the *CHARMM-GUI* feature (Jo *et al.*, 2008 ▸). *CHARMM* was chosen for its dedicated focus on macromolecules, making it particularly suitable for this study, as well as for its accessibility, being freely available in its basic form.

Corrections due to the chemical environment were made using the dipole interaction model described earlier. To achieve a more isotropic environment for GruPol calculations, a cutoff of 8 Å was applied. Essentially, this process involves creating an ellipsoid around the peptide, shrinking it to the smallest acceptable size that still encompasses the entire protein, and then enlarging the ellipsoid by the specified cutoff distance. For a detailed description of this approach, the reader is referred to our latest paper on GruPol functionalities (Ligorio *et al.*, 2024 ▸). Note that all the ions were kept after cutting the water box, in order to ensure proper charge balance in the entire system. Non-bonded ions exert a smaller influence due to the weaker electric field they produce on the protein, given their greater distance. This effect cannot be neglected and is indeed considered in GruPol, especially because other negative and positive sites are present. For example, even if the ions are not bound, such as when a sodium ion is near the oxygen atom of a peptide bond, they still have a potentially significant impact.

## 3. Results and discussion

### 3.1. *Ab initio* versus GruPol

**Figure 3:**
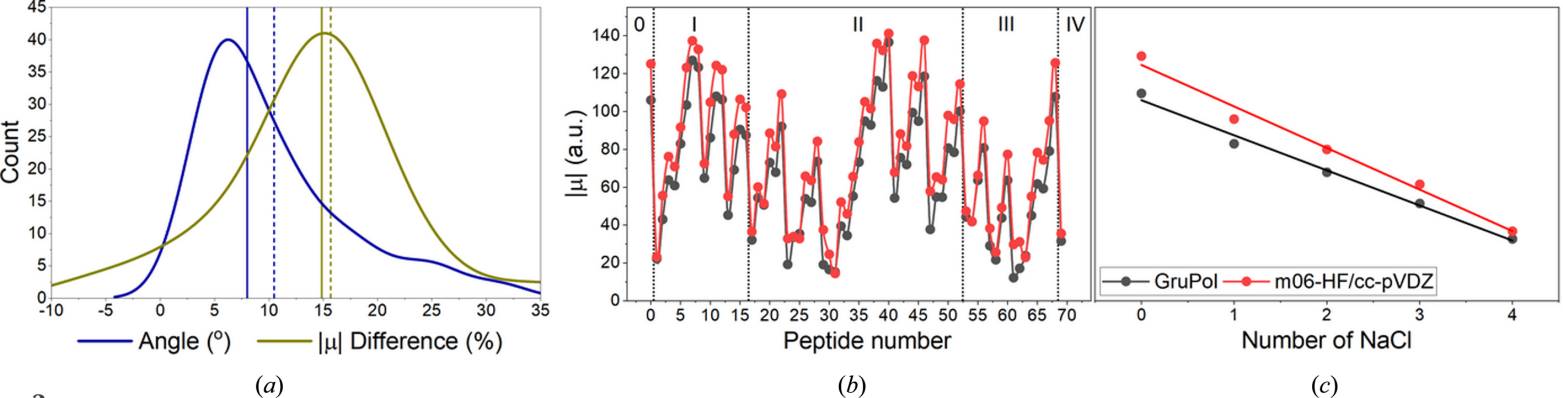
(*a*) Differences in angle and magnitude of dipole moments as predicted by GruPol and *ab initio* quantum calculations at the M06/cc-pVDZ level. The vertical solid lines represent the median deviations, while the dashed lines show the mean deviations. (*b*) Individual dipole moment magnitudes for each of the 70 calculations, varying from zero to four NaCl units within the protein backbone. (*c*) Average dipole moments as a function of the number of ions, with solid lines indicating the linear regression. The case involving zero ions bound was calculated assuming the terminal zwitterion state, where all other ionizable residues are in their neutral form.

Dipole moments calculated using GruPol closely align with those obtained from M06-HF/cc-pVDZ, with an average deviation of 15% in magnitude and 10° in angular direction, as shown in Fig. 3 ▸(*a*). This deviation is similar to the error observed with GruPol when previously benchmarking the database without ions (Ligorio *et al.*, 2024 ▸), indicating that the proposed model effectively corrects dipole moments influenced by ion presence. Fig. 3 ▸(*b*) depicts the significant fluctuations in dipole moments that are observed when ions are present, which are naturally dependent on their positions along the protein backbone. It is noteworthy that the average dipole moment, as shown in Fig. 3 ▸(*c*), systematically decreases with an increasing number of ions in the protein backbone. This trend can be attributed to the overall reduction in the number of charged sites and a more homogeneous distribution of charges around the protein backbone. Detailed numerical values for dipole moments and polarizabilities across all 70 configurations are provided in the supporting information, along with the corresponding numbering and naming based on ion positions.

Although the presence of ions bound to a given ionizable residue eliminates its contribution to the charge dipole moment, the significance of correcting the core dipole with these charges is demonstrated in Table 2 ▸. As shown, the salt BCDYZW (number 69), which includes three pairs of NaCl ions not associated with terminal groups, exhibits similar dipole moments to standard calculations involving only the terminal zwitterion state. This observation suggests that the presence of ions induces changes in the properties similar to hydrogen atoms in acidic groups, or neutralizes the extra hydrogen atom in basic groups. Despite the comparable dipole moments between the two scenarios (ions and H atoms), *ab initio* calculations show that the ion-containing species possess a slightly higher **μ**. This difference could also be predicted with GruPol. The core dipole correction is responsible for this effect, as simply removing the charges of the ionizable residues from **μ**_charge_ without taking into account **μ**_core_ would result in the same dipole moment as the standard calculation. GruPol was not only effective in correcting the magnitude of the dipole moment but also all of its components.

Before discussing the performance of the database to predict polarizabilities, it is important to highlight that GruPol was built using the M06-HF/aug-cc-pVDZ level of theory. Unfortunately, despite extensive efforts, we were unable to achieve SCF convergence for any of the peptides with this basis set, which is more accurate than its non-augmented counterpart, particularly for evaluating polarizabilities (Ligorio *et al.*, 2020 ▸). Yet, even with the less demanding cc-pVDZ basis set, convergence issues persisted for three peptides: B--Z--, B--W-- and BC-ZW-. To overcome these limitations, the YQC approach, which is implemented in *GAUSSIAN16*, was chosen. In short, the YQC method improves SCF convergence by starting with steepest descent steps and then switching to the regular SCF method, only using a more complex quadratic approach if needed. It is slower but more reliable than standard methods, especially for large molecules. Although the dipole moments obtained were consistent with those from other calculations, the polarizability values were exceedingly high and unrealistic, and consequently were discarded.

**Figure 4:**
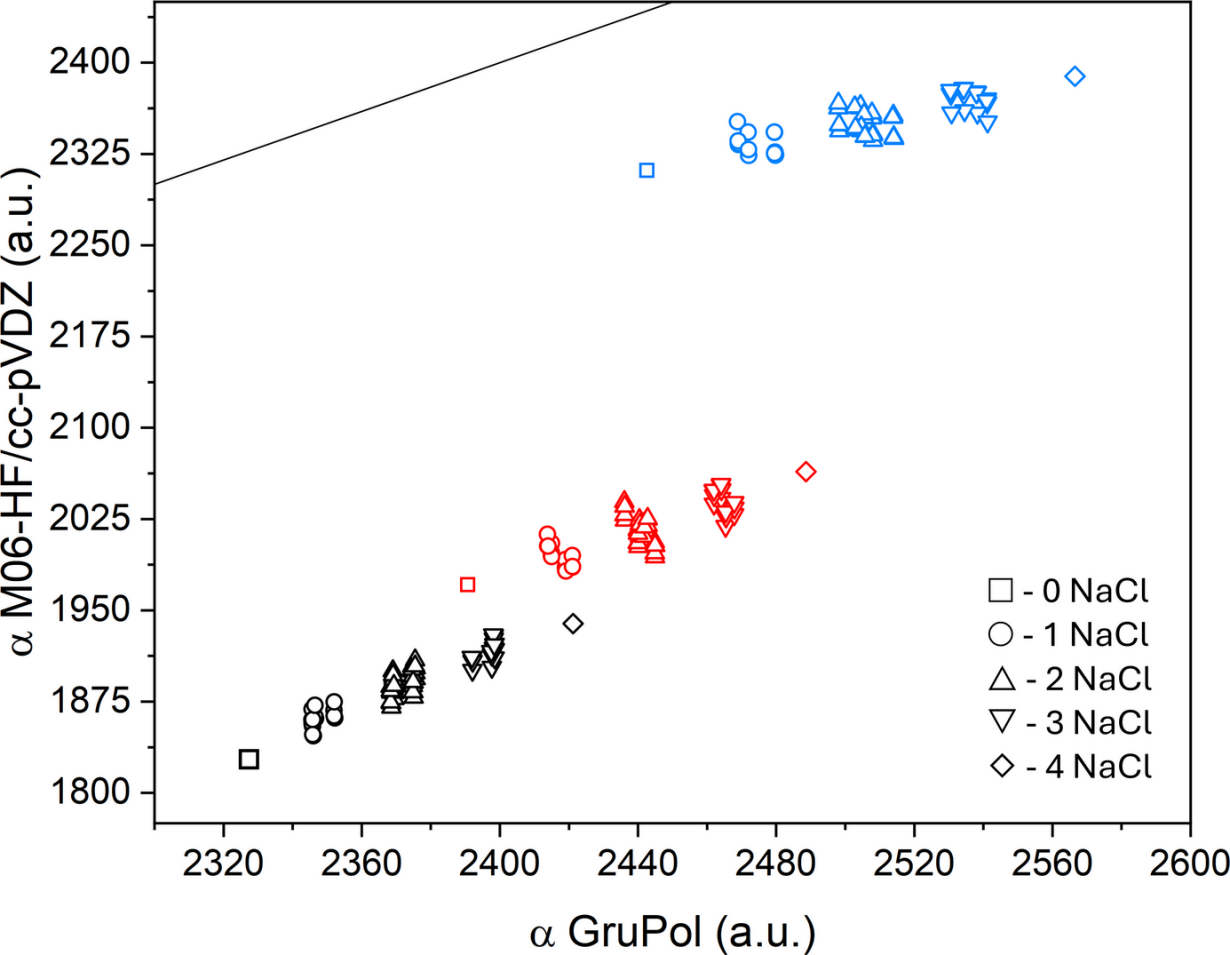
Comparison of the principal components of polarizabilities obtained from GruPol and *ab initio* quantum calculations at the M06-HF/cc-pVDZ level of theory. Black points represent α_11_, red points α_22_ and blue points α_33_. The solid line represents the ideal correspondence between the database and the quantum calculations.

**Figure 5:**
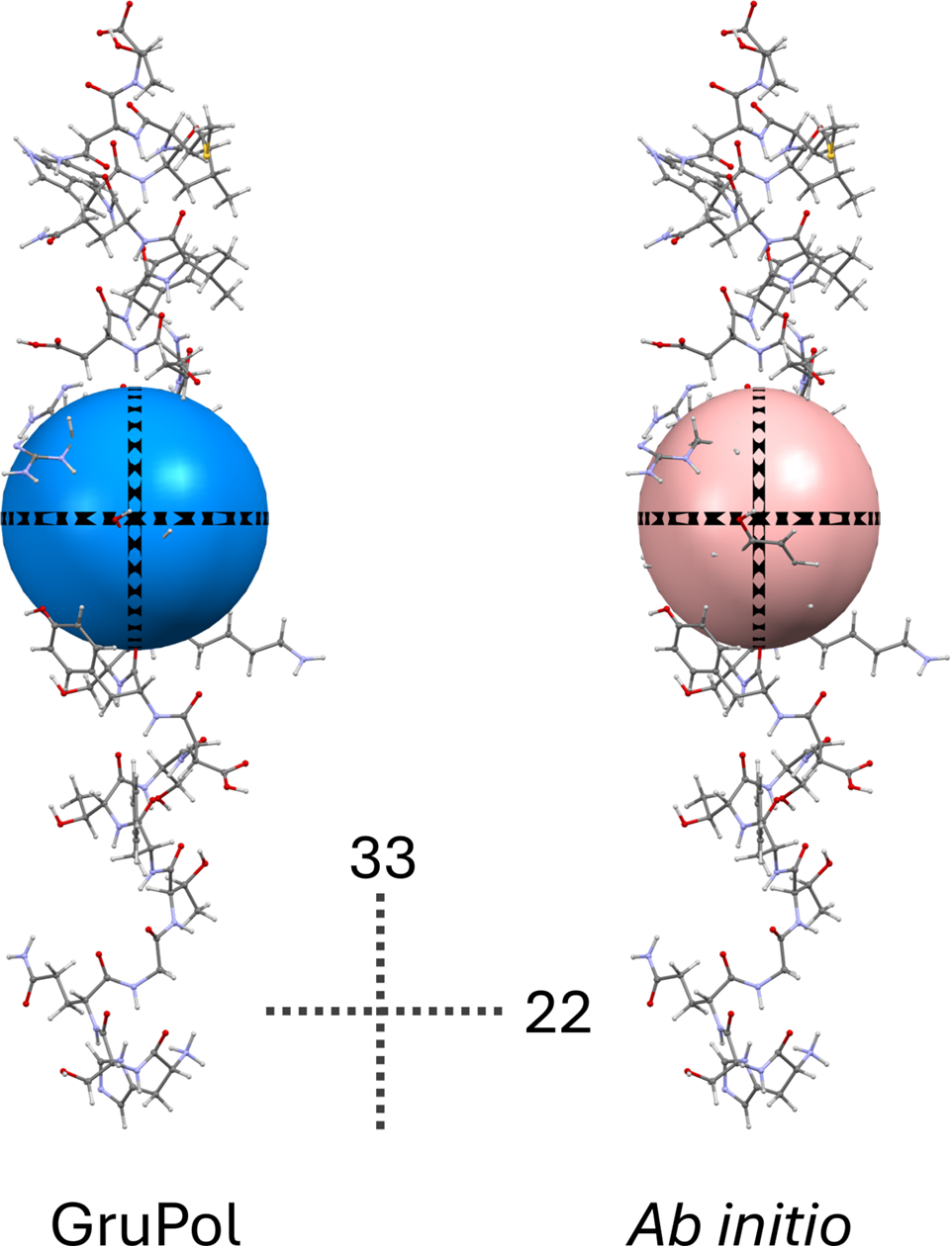
Molecular polarizabilities of glucagon obtained using standard GruPol and *ab initio* quantum calculations at the M06-HF/cc-pVDZ level (no ions coordinated). The polarizability tensor values have been reduced by a factor of 100. The labels 22 and 33 refer to the components of the polarizability tensor.

As anticipated, the principal components of polarizability tensors calculated using *ab initio* quantum methods exhibit values up to nearly 30% lower than those obtained with GruPol. However, a linear increase in these components could be predicted with the addition of ions, as illustrated in Fig. 4 ▸. While the α_11_ and α_22_ components show average deviations of approximately 20–25%, the α_33_ component exhibits a significantly lower deviation of 6–7%. This can be attributed to the approximate alignment of the α_33_ component with the mol­ecular axis of glucagon. The superposition of the atomic basis set in this direction creates an effect similar to that of diffuse functions. In contrast, components perpendicular to the helix are considerably lower due to the limited spatial coverage resulting from the absence of diffuse functions, as seen in Fig. 5 ▸.

### 3.2. Molecular dynamics simulation

**Figure 6:**
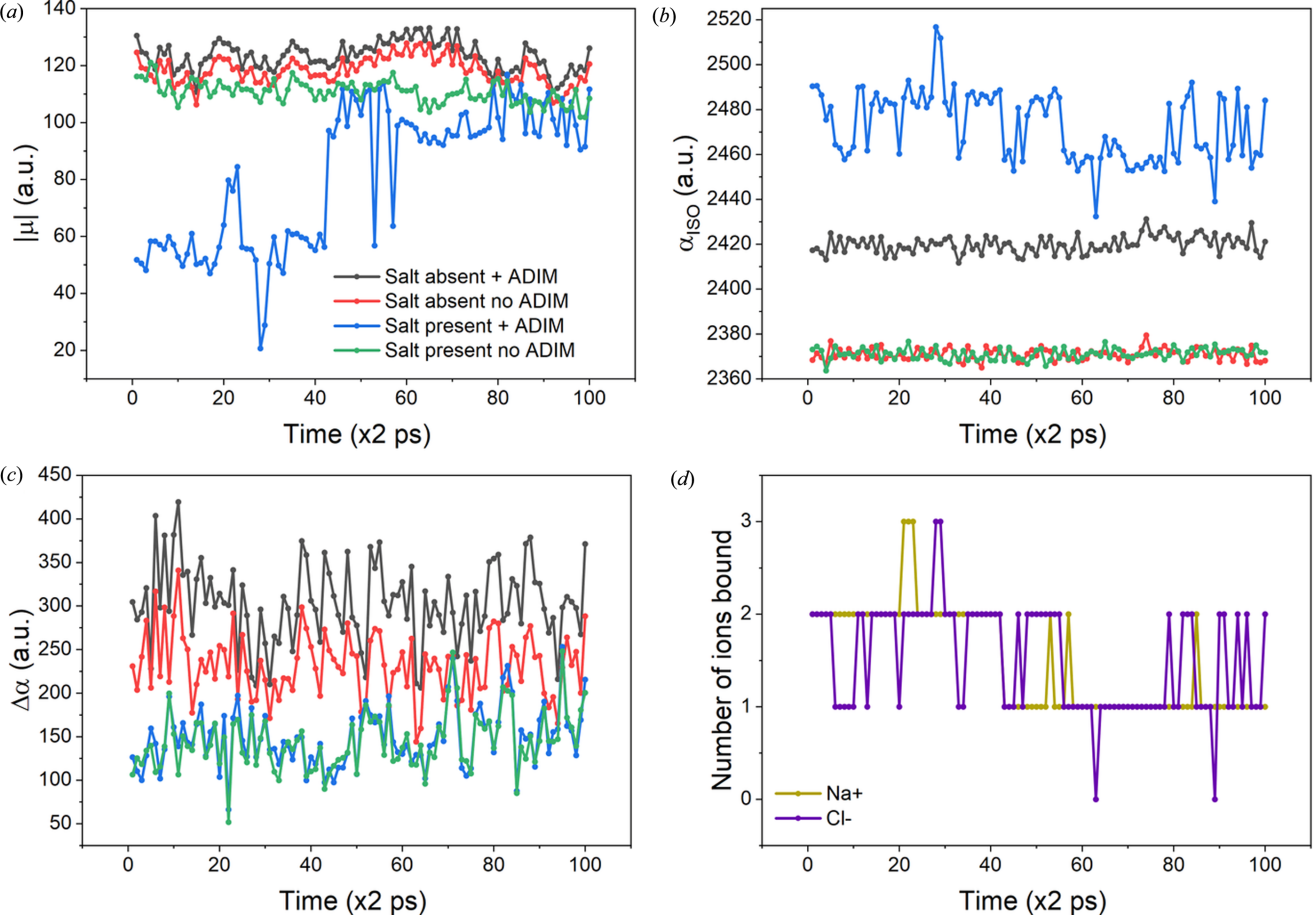
(*a*) Dipole moments, (*b*) isotropic polarizabilities, (*c*) anisotropy of polarizability and (*d*) number of ions coordinated to the protein’s backbone during the molecular dynamics simulation. Isotropic polarizability is defined as **α**_iso_ = Tr(α)/3 and anisotropy of polarizability is given by the formula Δα = 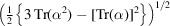.

To evaluate the properties obtained after molecular dynamics simulation, we first calculated polarizabilities and dipole moments for the different conformers of the protein without using ADIM and then employing the dipole inter­action model. This approach enabled us to isolate and understand the impact of intrinsic geometric variations on the electric properties, since the chemical environment is responsible for changing both the properties themselves and the molecular geometries. When evaluating the influence of the chemical environment in the ion-free simulation, the application of the interaction model led to an increase in dipole moments, as shown in Fig. 6 ▸(*a*). Note that, throughout the entire simulation, the curves with and without usage of the interaction model (black and red, respectively) can be almost superimposed, differing by a translation of 5–10 a.u.

The presence of ions decreased **μ**, either when ADIM was utilized or not, with the former situation presenting significantly lower values. These findings are consistent with the results visualized in Fig. 3 ▸(*c*), demonstrating that the presence of ions attached to ionizable residues may reduce the dipole moment. Interestingly, in the presence of ions, the dipole moment follows a distinct pattern: during the first half of the simulation, it was approximately 50% percent of the value observed in the latter half. This behaviour can be understood by the number of bound ions throughout the simulation, as shown in Fig. 6 ▸(*d*). Initially, an average of two pairs of NaCl were bound, which later decreased to one pair, corroborating the results observed previously. When analysing both curves without ADIM (red and green), a clear difference of 10–20 a.u. can be observed, suggesting that the simulations may have resulted in intrinsically distinct geometries due to the presence of ions.

Polarizability results showed that when ADIM was not employed, the isotropic values (**α**_iso_) remained nearly constant, with only minor fluctuations of a few atomic units. When the chemical environment was considered, the polarizability values increased by nearly 2% in the simulation without ions. In the presence of NaCl, the inclusion of ions, particularly chloride ones, significantly increased **α**, with values of around 30 a.u. for each ion bound. The presence of Na^+^ ions had a negligible effect on polarizability, given its isotropic value of 0.3 a.u., a consequence of the very contracted nature of its electron density. For this reason, the patterns observed in Figs. 6 ▸(*b*) and 6 ▸(*d*) are similar, particularly when comparing the blue curve in Fig. 6 ▸(*b*) (ADIM + ions) with the purple curve in Fig. 6 ▸(*d*) (number of chloride ions). In terms of polarizability anisotropy (Δ**α**), ADIM does not affect its value in the simulation with NaCl, as demonstrated in Fig. 6 ▸(*c*), where both curves overlap. In contrast, the simulation without ions produced higher Δ**α** values, regardless of whether ADIM was applied. Nevertheless, the chemical environment contributed to an increase in anisotropy compared to the ADIM-free scenario.

**Figure 7:**
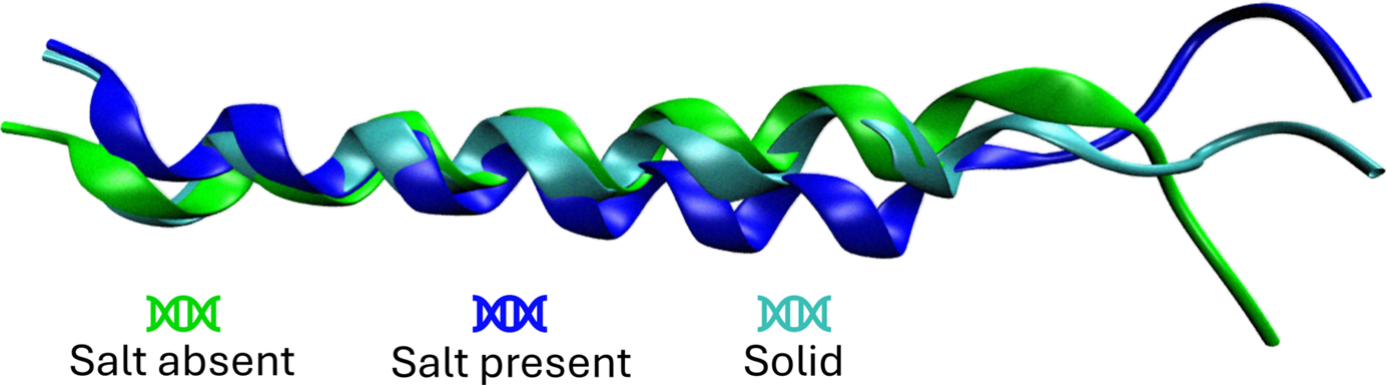
Representations of the glucagon molecule obtained at frame 29 from both molecular simulations and the solid-state structure.

A closer examination of the properties obtained, (i) for the molecule extracted from the experimental crystal structure (PDB 1gcn), *i.e.* the initial coordinates employed for the molecular dynamics simulations, and (ii) those derived from the simulations themselves, reveals that the presence of salt results in properties closely resembling those of the solid-state geometry. Fig. 7 ▸ provides a comparison of geometries across the three possible scenarios, with the snapshots taken from the simulation at 58 ps, corresponding to frame 29. The comparison of properties was conducted without the use of ADIM, focusing solely on the effects of geometric changes, exhibiting less fluctuation throughout the dynamics. For this reason, neither crystal field effects nor solvent were considered. Frame 29 was selected since at this frame, in the three scenarios, the properties were close to the average value across the entire simulation. These results indicate that the presence of salt stabilizes the protein, making it less susceptible to geometric changes during the simulation. Numerical values of the properties for the entire simulation are given in the supporting information; data only for one simulation step, frame 29, are given in Table 3 ▸.

## 4. Conclusions

Electrical charges on a protein backbone play a crucial role in accurately determining its electrical and optical properties, such as dipole moments and polarizabilities. For this reason, the GruPol database was developed with the goal of providing fast yet accurate predictions of these properties. The database enables the inclusion of charges in the backbone by selecting a specific pH, which then assigns charges to ionizable residues. In the presented update of the database, we have incorporated the option to include ions on the protein backbone, either as a salt in the solid state or within an aqueous environment.

To demonstrate the effects of salt and/or electrical charges in general, we selected the glucagon molecule and benchmarked its dipole moments and polarizabilities against *ab initio* quantum calculations, accounting for 70 different possibilities for ion arrangement along the protein’s backbone. We have showed that GruPol yields small errors for the dipole moment, averaging 15% in magnitude and 10° in angular direction. These error values are comparable with those obtained when benchmarking GruPol without ions or charges on the protein backbone. We observed that, in general, the more ions are linked to the protein, the lower its dipole moment tends to be, depending naturally on their specific positions on the protein structure. In the case of polarizability behaviour, GruPol exhibits a linear correlation with quantum calculations, although the database tends to result in higher values, primarily due to the absence of diffuse basis sets in the present quantum calculations. This discrepancy underscores the need to use the database when accurate properties are required, especially given the size limitations of proteins.

The second step of this study involved solvating the protein in two distinct aqueous media: one containing only the protein and water molecules, and the other including including Na^+^ and Cl^−^ ions as well. We observed that the inclusion of ions consistently reduced the overall dipole moment of the protein throughout the simulation, as well as the anisotropy of the polarizability. The reduced dipole moment can be attributed to two factors: (i) intrinsic geometric changes induced by the medium, and (ii) the presence of ions bound to the protein, which decreases the overall charge on the backbone. In simulations with ions present, the observed properties were more closely aligned with those from the solid-state structure, suggesting that the geometry was more stable and less prone to changes in the presence of salt.

## Supplementary Material

Tables S1-S5. DOI: 10.1107/S2052520625001088/pen5010sup1.pdf

## Figures and Tables

**Table 1:** p*K*_a_ values used to calculate charges of ionizable residues CTER and NTER refer to the terminal COO^−^ and NH_3_^+^ groups, respectively.

Group	Charge	p*K*_a_
GLU/ASP	−	4.4
CYS	−	8.5
TYR	−	10.1
ARG	+	12.5
LYS	+	10.6
HIS	+	6.6
CTER	+	4.0
NTER	−	8.0

**Table 2:** Comparison of dipole moments obtained using GruPol and *ab initio* quantum calculations for peptide 69, BCDYZW (which includes three pairs of coordinated ions, excluding the terminal groups), with those from a standard GruPol calculation, peptide 1 Results are given in atomic units (a.u.).

	GruPol				M06-HF/cc-pVDZ			
	μ_*x*_	μ_*y*_	μ_*z*_	|μ|	μ_*x*_	μ_*y*_	μ_*z*_	|μ|
BCDYZW (69)	106.9	12.9	−4.9	107.8	123.7	22.3	−1.7	125.7
Standard (1)	105.3	11.1	−5.8	106.0	123.4	20.6	−4.6	125.2

**Table 3:** Polarizabilities and dipole moments calculated for glucagon using the solid-state geometry and at frame 29 (58 ps) extracted from simulations conducted both with and without the presence of NaCl In this analysis, the dipole interaction model was not applied, allowing us to focus on the changes in these properties resulting solely from intrinsic geometric variations across these three scenarios. Despite the absence of environmental interactions via ADIM, charges were included on the protein backbone, assuming a pH of 7.3.

	**α**_iso_	Δ**α**	|μ|
Salt absent	2369.2	237.3	117.2
Salt present	2368.8	148.4	111.5
Solid	2386.9	102.0	94.3
