# Supplementary material for: Electric charge and salting in/out effects on glucagon’s dipole moments and polarizabilities using the GruPol database
Source: Acta Crystallogr B Struct Sci Cryst Eng Mater. 2025 Feb 24;81(Pt 2):192–201. doi: 10.1107/S2052520625001088 (PMC11970118; doi:10.1107/S2052520625001088)
Supplement: Supplementary file 1 [file b-81-00192-sup1.pdf]

STRUCTURAL SCIENCE
CRYSTAL ENGINEERING
MATERIALS

Volume 81 (2025)

Supporting information for article:

Electric charge and salting in/out effects on glucagon's dipole moments and polarizabilities using the GruPol database

Raphael F. Ligorio, Rasmus H. M. Gehle, Leonardo H. R. dos Santos and Anna Krawczuk

S1 GruPol results for the glutamide amidotransferase

Table S1: Polarizabilities and dipole moments (a.u.) for the four chains of the glutamide aminotransferase molecule, calculated independently using GruPol applying a pH of 7.3.

Chain	μ_X	μ_Y	μ_Z	α_{xx}	α_{yy}	α_{zz}	α_{xy}	α_{xz}	α_{yz}	α_{iso}
A	138.39	-61.52	-8.25	33618.6	35201.9	33162.7	-117.0	75.9	-128.6	33994.4
B	135.17	21.38	10.36	33927.9	34971.3	33145.2	441.2	-76.0	-94.2	34014.8
C	-142.31	56.80	-15.30	33639.2	35166.3	33189.6	-124.6	-65.9	103.2	33998.4
D	-148.82	-22.34	-6.23	33826.4	34988.8	33159.4	543.5	142.3	120.5	33991.5

S2 Glucagon benchmarking

Table S2: GruPol polarizabilities and dipole moments (a.u.) for the glucagon peptide in the presence of NaCl.

Number	Code	α_{11}	α_{22}	α_{33}	Mu_X	Mu_Y	Mu_Z
1	STANDARD	2327.5	2390.8	2442.6	105.30	11.09	-5.84
2	A _ _ X _ _	2352.2	2419.0	2468.8	20.67	2.05	-7.95
3	A _ _ Y _ _	2345.8	2414.9	2479.5	37.80	14.83	-14.21
4	A _ _ Z _ _	2345.9	2413.8	2479.4	62.53	12.94	1.31
5	A _ _ W _ _	2346.6	2421.0	2471.8	60.01	-1.74	-10.08
6	B _ _ X _ _	2352.0	2419.0	2469.1	82.55	7.30	-4.39
7	B _ _ Y _ _	2345.6	2414.9	2479.8	100.82	20.10	-11.41
8	B _ _ Z _ _	2345.7	2413.8	2479.7	125.61	18.26	4.10
9	B _ _ W _ _	2346.4	2420.9	2472.1	123.08	3.58	-7.28
10	C _ _ X _ _	2352.0	2419.0	2469.0	63.28	-0.60	-14.58
11	C _ _ Y _ _	2345.6	2414.9	2479.7	82.76	12.68	-20.63
12	C _ _ Z _ _	2345.8	2413.8	2479.7	107.44	10.79	-5.20
13	C _ _ W _ _	2346.4	2421.0	2472.0	104.94	-3.86	-16.54
14	D _ _ X _ _	2352.0	2419.1	2468.9	43.40	6.53	-11.36
15	D _ _ Y _ _	2345.6	2415.0	2479.6	64.10	19.22	-17.69

16	D _ _ Z _ _	2345.8	2413.9	2479.5	88.85	17.31	-2.22
17	D _ _ W _ _	2346.4	2421.0	2472.0	86.40	2.72	-13.54
18	A B _ X Y _	2374.8	2440.0	2504.6	29.50	11.40	-5.70
19	A B _ X Z _	2375.0	2440.4	2502.9	52.94	9.73	8.65
20	A B _ X W _	2375.6	2444.9	2498.1	50.49	-3.98	-2.07
21	A B _ Y Z _	2368.6	2436.1	2513.9	69.42	22.68	2.54
22	A B _ Y W _	2369.2	2441.8	2507.9	66.89	7.99	-8.85
23	A B _ Z W _	2369.4	2442.7	2505.6	91.69	6.16	6.68
24	A C _ X Y _	2374.8	2440.0	2504.5	10.31	3.54	-15.84
25	A C _ X Z _	2375.0	2440.5	2502.8	33.62	1.82	-1.58
26	A C _ X W _	2375.6	2445.0	2498.0	31.21	-11.87	-12.24
27	A C _ Y Z _	2368.6	2436.1	2513.9	51.33	15.26	-6.73
28	A C _ Y W _	2369.2	2441.8	2507.8	48.84	0.60	-18.06
29	A C _ Z W _	2369.5	2442.8	2505.5	73.51	-1.29	-2.62
30	A D _ X Y _	2374.8	2440.1	2504.4	-9.57	10.68	-12.65
31	A D _ X Z _	2375.0	2440.6	2502.7	13.82	8.93	1.65
32	A D _ X W _	2375.6	2444.9	2498.0	11.45	-4.69	-9.00
33	A D _ Y Z _	2368.6	2436.2	2513.8	32.73	21.78	-3.77
34	A D _ Y W _	2369.2	2441.9	2507.7	30.29	7.19	-15.09
35	A D _ Z W _	2369.5	2442.8	2505.4	55.04	5.27	0.39
36	B C _ X Y _	2374.6	2440.0	2504.8	71.91	8.29	-11.88
37	B C _ X Z _	2374.8	2440.4	2503.1	94.64	6.70	1.85
38	B C _ X W _	2375.4	2444.9	2498.3	92.27	-6.54	-8.51
39	B C _ Y Z _	2368.4	2436.0	2514.2	114.34	20.52	-3.94
40	B C _ Y W _	2369.0	2441.7	2508.1	111.84	5.86	-15.28
41	B C _ Z W _	2369.3	2442.7	2505.8	136.58	4.03	0.17
42	B D _ X Y _	2374.6	2440.0	2504.7	51.37	15.75	-8.55
43	B D _ X Z _	2374.8	2440.5	2503.0	74.19	14.14	5.22
44	B D _ X W _	2375.4	2444.9	2498.3	71.85	0.96	-5.13
45	B D _ Y Z _	2368.4	2436.1	2514.1	95.75	27.04	-0.98
46	B D _ Y W _	2369.0	2441.8	2508.0	93.30	12.45	-12.31
47	B D _ Z W _	2369.3	2442.8	2505.7	118.11	10.59	3.18

48	C D _ X Y _	2374.7	2440.1	2504.6	31.58	7.66	-19.21
49	C D _ X Z _	2374.9	2440.5	2503.0	54.27	5.99	-5.53
50	C D _ X W _	2375.5	2444.9	2498.2	51.98	-7.15	-15.83
51	C D _ Y Z _	2368.4	2436.2	2514.0	77.66	19.62	-10.24
52	C D _ Y W _	2369.1	2441.8	2507.9	75.25	5.06	-21.52
53	C D _ Z W _	2369.3	2442.8	2505.7	99.94	3.15	-6.11
54	A B C X Y Z	2397.7	2461.9	2538.1	43.06	10.65	0.70
55	A B C X Y W	2398.2	2465.4	2534.3	40.68	-2.60	-9.66
56	A B C X Z W	2398.5	2467.8	2530.6	63.43	-4.19	4.08
57	A B C Y Z W	2392.0	2464.0	2541.1	80.38	8.44	-1.37
58	A B D X Y Z	2397.7	2462.0	2538.0	22.60	18.08	4.04
59	A B D X Y W	2398.3	2465.4	2534.4	20.27	4.91	-6.31
60	A B D X Z W	2398.5	2467.9	2530.6	43.08	3.29	7.47
61	A B D Y Z W	2392.1	2464.1	2541.0	61.91	15.01	1.61
62	A C D X Y Z	2397.7	2462.1	2538.0	2.76	9.99	-6.66
63	A C D X Y W	2398.3	2465.4	2534.3	0.47	-3.16	-16.96
64	A C D X Z W	2398.5	2467.9	2530.5	23.17	-4.83	-3.27
65	A C D Y Z W	2392.1	2464.1	2540.9	43.82	7.61	-7.63
66	B C D X Y Z	2397.5	2462.0	2538.3	60.20	13.84	-2.67
67	B C D X Y W	2398.1	2465.4	2534.5	58.00	1.89	-12.14
68	B C D X Z W	2398.3	2467.8	2530.8	79.07	0.49	0.10
69	B C D Y Z W	2391.9	2464.1	2541.2	106.94	12.89	-4.86
70	ABCDXYZW	2421.2	2488.6	2566.5	31.40	4.04	-0.72

Table S3: *Ab initio* polarizabilities and dipole moments (a.u., M06-HF/cc-pVDZ) for the glucagon peptide in the presence of NaCl.

Number	Code	α_{11}	α_{22}	α_{33}	Mu_X	Mu_Y	Mu_Z
1	STANDARD	1827.4	1970.8	2311.3	123.36	20.56	-4.56
2	A _ _ X _ _	1861.6	1983.0	2351.7	18.16	11.71	-8.44
3	A _ _ Y _ _	1855.0	1995.0	2342.9	47.73	22.72	-17.49
4	A _ _ Z _ _	1846.9	2003.0	2343.1	72.39	20.23	12.75

5	A _ _ W _ _	1861.3	1985.6	2343.1	70.84	1.70	-2.63
6	B _ _ X _ _	1868.0	1985.6	2332.8	89.81	18.34	-0.92
7	B _ _ Y _ _	1860.1	1997.7	2324.5	119.32	29.31	-9.97
8	B _ _ Z _ _	—	—	—	133.33	25.44	20.73
9	B _ _ W _ _	—	—	—	132.52	7.01	5.32
10	C _ _ X _ _	1863.3	1991.8	2333.6	68.47	12.05	-20.64
11	C _ _ Y _ _	1857.0	2005.4	2324.2	98.04	23.05	-29.65
12	C _ _ Z _ _	1848.0	2012.5	2324.8	122.64	20.55	0.45
13	C _ _ W _ _	1862.9	1995.1	2324.0	121.12	2.05	-14.84
14	D _ _ X _ _	1874.9	1982.4	2335.9	48.17	20.24	-17.94
15	D _ _ Y _ _	1869.0	1994.4	2326.6	77.75	31.24	-26.98
16	D _ _ Z _ _	1860.3	2002.9	2325.6	102.40	28.75	3.21
17	D _ _ W _ _	1872.0	1986.0	2328.7	100.91	10.31	-12.10
18	A B _ X Y _	1890.2	2006.0	2362.8	27.90	22.11	-8.46
19	A B _ X Z _	1882.9	2015.1	2363.0	52.59	19.65	21.77
20	A B _ X W _	1898.4	1997.0	2363.1	51.03	1.13	6.39
21	A B _ Y Z _	1873.8	2028.3	2356.8	82.10	30.63	12.73
22	A B _ Y W _	1888.9	2010.3	2356.2	80.55	12.10	-2.66
23	A B _ Z W _	1882.1	2019.2	2355.5	105.24	9.64	27.59
24	A C _ X Y _	1887.8	2013.7	2362.7	6.60	15.84	-28.12
25	A C _ X Z _	1878.4	2020.7	2363.3	31.21	13.35	1.97
26	A C _ X W _	1893.6	2003.0	2362.7	29.69	-5.15	-13.32
27	A C _ Y Z _	1870.3	2035.2	2354.9	60.78	24.36	-7.03
28	A C _ Y W _	1885.6	2017.9	2354.5	59.27	5.85	-22.32
29	A C _ Z W _	1877.3	2024.7	2354.8	83.88	3.36	7.78
30	A D _ X Y _	1899.9	2002.3	2364.6	-13.71	24.03	-25.46
31	A D _ X Z _	1890.8	2010.9	2363.5	10.95	21.55	4.72
32	A D _ X W _	1902.5	1993.7	2366.7	9.47	3.11	-10.59
33	A D _ Y Z _	1882.6	2023.9	2355.2	40.53	32.55	-4.32
34	A D _ Y W _	1894.7	2006.5	2358.6	39.04	14.12	-19.63
35	A D _ Z W _	1887.3	2015.4	2357.8	63.70	11.62	10.57
36	B C _ X Y _	1891.2	2016.3	2344.0	78.22	22.43	-20.62

37	B C _ X Z _	1883.0	2024.4	2344.7	102.85	19.97	9.47
38	B C _ X W _	1898.9	2006.2	2343.9	101.33	1.47	-5.83
39	B C _ Y Z _	1874.5	2039.2	2337.7	132.37	30.94	0.48
40	B C _ Y W _	1889.9	2021.3	2336.0	130.85	12.43	-14.82
41	B C _ Z W _	—	—	—	140.06	7.94	15.96
42	B D _ X Y _	1903.9	2005.3	2346.3	57.92	30.63	-17.95
43	B D _ X Z _	1896.0	2014.9	2345.5	82.60	28.17	12.23
44	B D _ X W _	1909.1	1997.1	2348.8	81.11	9.74	-3.09
45	B D _ Y Z _	1887.3	2028.5	2339.1	112.12	39.14	3.20
46	B D _ Y W _	1900.2	2010.5	2341.6	110.63	20.70	-12.12
47	B D _ Z W _	1893.1	2019.7	2339.3	135.30	18.24	18.08
48	C D _ X Y _	1901.5	2013.1	2347.2	36.61	24.37	-37.61
49	C D _ X Z _	1891.4	2020.6	2346.5	61.21	21.88	-7.58
50	C D _ X W _	1904.2	2003.4	2349.1	59.77	3.47	-22.80
51	C D _ Y Z _	1883.8	2035.4	2338.3	90.79	32.88	-16.56
52	C D _ Y W _	1896.9	2018.3	2340.4	89.34	14.47	-31.79
53	C D _ Z W _	1888.2	2025.5	2339.5	113.94	11.97	-1.73
54	A B C X Y Z	1903.4	2048.3	2375.7	40.96	23.74	1.99
55	A B C X Y W	1919.0	2030.5	2374.3	39.44	5.24	-13.31
56	A B C X Z W	1911.2	2038.1	2374.2	64.07	2.78	16.79
57	A B C Y Z W	1900.9	2052.9	2367.2	93.59	13.74	7.80
58	A B D X Y Z	1916.3	2037.4	2376.2	20.69	31.94	4.70
59	A B D X Y W	1929.3	2019.2	2379.2	19.21	13.51	-10.61
60	A B D X Z W	1922.3	2028.7	2377.3	43.89	11.05	19.58
61	A B D Y Z W	1911.3	2042.1	2370.4	73.41	22.01	10.54
62	A C D X Y Z	1913.3	2044.4	2375.5	-0.65	25.67	-15.05
63	A C D X Y W	1926.6	2027.0	2378.8	-2.10	7.26	-30.27
64	A C D X Z W	1918.0	2034.2	2377.3	22.51	4.77	-0.22
65	A C D Y Z W	1908.3	2049.3	2368.9	52.09	15.77	-9.21
66	B C D X Y Z	1916.3	2048.8	2358.6	70.97	32.26	-7.54
67	B C D X Y W	1930.0	2030.9	2360.6	69.52	13.85	-22.77
68	B C D X Z W	1921.7	2038.7	2358.8	94.14	11.39	7.28

69	B C D Y Z W	1911.5	2053.7	2351.5	123.66	22.34	-1.71
70	ABCDXYZW	1939.2	2064.0	2388.9	32.25	15.15	-0.19

S3 Glucagon molecular dynamics

Table S4: GruPol polarizabilities and dipole moments (a.u.) for the glucagon peptide throughout the molecular dynamics simulation in the absence of NaCl

	No salt + ADIM			No Salt - No ADIM		
Frame	α_{iso}	$\Delta\alpha$	$\|\mu\|$	α_{iso}	$\Delta\alpha$	$\|\mu\|$
1	2417.4	304.6	130.54	2368.4	230.9	124.62
2	2418.1	284.5	124.81	2371.4	203.7	119.28
3	2416.1	292.9	124.19	2369.6	241.7	118.82
4	2413.1	320.7	120.84	2366.7	283.4	116.60
5	2425.0	228.3	118.75	2376.9	206.2	114.35
6	2416.9	403.7	126.33	2369.5	316.9	121.64
7	2423.3	298.3	124.03	2373.1	219.2	117.85
8	2416.5	381.1	127.01	2369.5	298.5	121.83
9	2422.5	294.0	116.82	2373.4	212.9	111.97
10	2421.5	381.9	118.62	2370.6	288.6	113.57
11	2419.1	419.5	119.85	2369.0	341.0	114.41
12	2422.3	335.7	123.71	2373.3	263.2	117.44
13	2418.7	339.5	117.62	2369.0	250.3	112.39
14	2422.9	266.8	110.71	2374.1	177.4	106.30
15	2416.3	330.7	120.46	2369.4	210.1	114.93
16	2423.3	355.4	122.30	2375.2	238.3	117.03
17	2413.8	303.4	123.19	2369.0	224.5	117.20
18	2418.6	332.5	127.76	2370.5	246.8	122.05
19	2414.0	299.1	129.56	2369.1	216.5	123.15
20	2419.6	314.3	127.84	2371.2	254.4	122.26
21	2418.0	303.6	127.71	2369.0	249.8	122.22
22	2418.3	300.9	125.43	2368.8	219.0	118.66

23	2415.8	341.4	125.74	2369.6	291.6	119.44
24	2422.3	226.6	118.84	2373.9	192.8	114.60
25	2420.0	324.0	123.32	2370.6	267.1	117.57
26	2417.6	289.3	124.61	2368.9	225.3	118.98
27	2421.5	218.0	119.80	2373.1	190.0	114.02
28	2420.1	208.7	119.22	2371.9	192.0	114.10
29	2420.1	296.1	122.90	2369.2	237.4	117.17
30	2422.2	257.0	118.70	2372.9	215.1	113.15
31	2423.3	210.2	117.73	2374.9	171.2	113.18
32	2418.5	265.2	120.93	2371.7	214.2	116.28
33	2411.7	257.4	123.55	2367.7	192.0	118.52
34	2415.9	311.0	125.23	2366.4	217.1	119.58
35	2423.3	297.3	128.51	2374.6	216.5	122.49
36	2417.6	248.0	126.52	2369.9	203.4	120.88
37	2419.8	289.7	125.19	2368.0	240.4	119.22
38	2415.1	374.8	125.22	2365.0	298.8	119.37
39	2423.7	358.6	121.12	2374.6	274.4	116.11
40	2419.9	306.1	121.39	2371.6	253.2	116.58
41	2423.0	295.8	121.67	2373.3	228.2	116.63
42	2416.5	258.8	121.48	2371.7	196.7	116.77
43	2420.2	361.5	119.20	2369.4	273.1	114.38
44	2420.7	337.5	119.99	2373.1	249.2	114.67
45	2417.1	311.6	122.41	2370.7	238.8	117.03
46	2413.8	289.7	128.40	2367.1	230.1	122.58
47	2413.3	270.2	121.70	2367.4	222.6	116.65
48	2419.7	362.5	126.34	2371.1	280.3	120.52
49	2418.1	287.1	123.44	2372.9	245.4	118.15
50	2414.9	277.8	124.73	2370.3	242.4	120.82
51	2423.4	246.1	126.45	2374.7	178.7	122.60
52	2418.9	218.1	126.10	2372.9	185.4	122.57
53	2423.4	368.1	125.15	2372.3	260.4	120.13
54	2414.7	343.4	130.28	2367.1	273.8	124.96

55	2420.0	373.4	127.69	2370.0	271.3	122.54
56	2419.5	305.4	127.78	2371.1	232.3	122.41
57	2414.1	289.0	131.19	2368.5	184.5	126.81
58	2417.8	313.0	129.09	2369.0	223.4	124.06
59	2425.2	312.3	129.74	2373.0	227.3	123.81
60	2414.4	327.7	132.73	2368.3	247.0	127.82
61	2415.0	285.2	129.30	2369.0	207.4	123.45
62	2420.0	345.3	132.81	2372.4	262.5	126.99
63	2417.4	211.3	132.97	2371.2	144.2	127.73
64	2417.5	206.2	128.84	2371.6	159.7	123.94
65	2419.6	317.0	133.22	2371.7	244.8	127.56
66	2417.2	277.5	126.44	2369.9	226.6	120.45
67	2422.9	304.6	127.19	2370.9	239.8	121.76
68	2417.4	289.7	129.46	2369.0	227.3	124.08
69	2424.6	270.0	132.92	2372.2	192.5	127.24
70	2418.9	333.9	125.33	2367.3	242.0	120.32
71	2419.8	292.6	132.85	2370.0	242.7	126.88
72	2419.6	242.1	125.84	2370.4	185.7	120.52
73	2426.0	279.4	122.85	2374.2	192.0	117.39
74	2431.2	312.3	123.26	2379.4	243.8	118.37
75	2424.1	237.2	128.39	2372.2	180.9	123.37
76	2422.6	316.6	122.89	2370.4	239.5	119.13
77	2425.4	271.2	121.36	2372.8	205.4	117.32
78	2427.6	288.3	117.47	2374.8	206.7	114.69
79	2423.4	350.9	115.28	2372.4	274.9	113.20
80	2421.8	354.5	122.14	2371.9	282.4	117.91
81	2424.8	359.0	117.25	2372.0	280.1	114.09
82	2415.9	283.9	116.46	2367.6	202.6	113.27
83	2420.9	291.4	118.03	2367.7	209.6	114.78
84	2421.7	331.0	119.31	2370.2	253.2	115.78
85	2425.4	323.3	117.84	2374.3	243.3	114.04
86	2426.2	279.8	127.86	2374.0	213.7	122.56

87	2423.0	371.5	125.33	2370.3	264.0	120.39
88	2419.4	378.9	124.96	2370.2	277.1	120.08
89	2425.0	327.1	120.80	2373.5	241.2	115.60
90	2414.6	325.7	121.52	2367.4	243.0	116.12
91	2422.1	296.5	116.22	2372.6	199.8	112.77
92	2424.0	269.4	110.96	2371.4	183.7	106.93
93	2418.9	286.5	112.06	2367.9	195.7	107.59
94	2422.7	216.0	113.53	2373.2	165.2	108.17
95	2420.8	298.5	115.98	2371.7	229.4	110.34
96	2418.3	310.6	120.16	2366.6	264.0	112.87
97	2429.6	304.9	116.86	2374.9	232.1	111.25
98	2417.2	297.7	119.59	2367.6	247.6	115.77
99	2414.2	267.5	118.26	2367.3	200.2	114.66
100	2421.2	371.1	126.11	2368.1	288.3	120.55

Table S5: GruPol polarizabilities and dipole moments (a.u.) for the glucagon peptide throughout the molecular dynamics simulation in the presence of NaCl

	Salt + ADIM			Salt - No ADIM		
Frame	α_{iso}	$\Delta\alpha$	$\|\mu\|$	α_{iso}	$\Delta\alpha$	$\|\mu\|$
1	2490.4	126.6	51.80	2373.1	106.5	116.20
2	2490.6	110.4	50.54	2374.5	125.4	116.19
3	2486.5	100.2	48.16	2372.5	118.6	115.00
4	2475.4	128.2	58.34	2363.7	134.1	121.14
5	2481.3	159.6	58.30	2369.2	139.7	119.87
6	2464.4	142.2	57.15	2374.6	109.3	110.96
7	2462.8	101.9	55.59	2370.7	115.6	109.77
8	2457.8	135.4	59.94	2371.0	138.8	114.39
9	2460.3	196.0	57.19	2371.3	199.7	110.29
10	2463.4	161.0	52.85	2369.8	153.2	105.37
11	2489.9	138.8	49.60	2371.8	106.4	109.14
12	2490.3	165.7	53.84	2374.2	150.6	112.59

13	2461.7	143.3	61.01	2372.3	139.1	115.70
14	2482.4	141.1	50.26	2370.1	134.5	114.92
15	2487.4	164.5	50.73	2374.8	166.3	112.61
16	2479.4	187.2	52.20	2367.7	165.8	114.05
17	2484.3	127.8	47.04	2371.8	126.5	109.11
18	2482.8	155.4	50.34	2371.1	140.1	110.81
19	2482.3	164.7	56.24	2368.7	165.2	114.63
20	2460.3	103.8	64.04	2372.9	119.2	112.33
21	2485.3	174.0	79.75	2371.6	149.3	112.06
22	2493.0	66.4	75.99	2376.6	52.0	109.68
23	2483.4	171.5	84.42	2369.0	164.6	113.54
24	2481.3	197.2	56.23	2369.0	169.6	111.58
25	2489.8	145.5	55.65	2373.5	131.6	111.74
26	2480.9	127.1	55.47	2370.2	120.5	111.31
27	2485.5	183.0	51.74	2369.8	175.0	109.23
28	2516.8	126.8	20.68	2374.8	117.5	107.18
29	2511.9	147.1	28.82	2368.8	148.4	111.53
30	2483.3	174.0	50.47	2367.4	167.8	111.31
31	2477.7	135.3	59.83	2366.7	131.1	115.32
32	2491.4	136.2	49.82	2372.1	109.6	108.46
33	2458.5	118.6	47.12	2368.7	99.9	106.69
34	2465.5	144.1	61.93	2372.6	133.9	111.53
35	2486.4	140.0	60.72	2368.3	143.9	117.43
36	2487.8	123.7	60.99	2371.8	135.8	114.93
37	2482.5	149.5	59.75	2368.2	146.1	113.48
38	2486.7	149.5	59.15	2369.3	156.4	113.07
39	2485.9	99.8	56.61	2371.4	105.0	111.44
40	2482.9	126.5	55.15	2368.2	110.1	107.95
41	2487.3	118.6	60.71	2368.2	112.8	111.00
42	2488.7	142.0	56.26	2374.0	137.4	108.27
43	2457.6	97.5	97.19	2368.0	89.9	111.02
44	2461.7	112.5	95.07	2374.0	107.0	109.80

45	2452.7	97.5	100.90	2368.5	116.5	113.71
46	2480.8	114.6	111.81	2369.5	123.6	113.36
47	2456.9	114.4	98.71	2368.6	125.8	112.11
48	2477.3	131.3	110.86	2366.6	131.9	114.10
49	2483.6	171.0	107.93	2369.7	163.6	110.92
50	2485.5	106.9	102.65	2372.8	107.3	108.05
51	2484.4	172.3	110.79	2373.4	158.4	113.30
52	2477.6	190.9	111.52	2365.7	186.2	113.16
53	2485.7	174.8	56.76	2368.3	167.0	111.48
54	2489.2	166.4	111.61	2373.5	173.3	113.90
55	2485.3	173.6	114.08	2369.2	166.9	114.65
56	2461.8	140.9	104.15	2374.3	129.0	114.32
57	2456.6	196.5	63.66	2369.0	186.3	117.50
58	2460.1	144.0	98.94	2372.2	122.1	112.56
59	2452.7	132.2	101.01	2367.7	124.5	111.30
60	2456.3	130.2	99.98	2369.5	137.4	111.16
61	2459.1	140.6	99.23	2370.9	153.0	111.30
62	2458.5	121.3	97.97	2372.2	118.1	112.61
63	2432.4	129.3	93.58	2371.4	117.8	104.57
64	2458.5	128.9	95.96	2370.1	140.0	108.04
65	2468.0	102.1	92.78	2376.5	96.0	103.71
66	2459.8	139.5	94.79	2369.5	131.8	107.75
67	2466.2	141.6	92.84	2374.2	126.6	105.37
68	2463.2	164.3	92.13	2373.2	161.0	106.44
69	2459.5	145.0	97.29	2370.3	151.0	109.52
70	2453.0	207.1	95.35	2370.0	202.9	110.02
71	2452.8	239.4	95.49	2369.2	246.9	110.32
72	2455.3	191.2	102.68	2370.8	205.9	110.89
73	2453.8	114.0	103.55	2370.4	123.8	115.17
74	2456.4	105.1	94.98	2371.1	122.2	108.58
75	2458.9	113.7	95.47	2371.5	107.6	108.18
76	2454.5	176.1	96.38	2373.0	175.2	109.48

77	2458.7	188.1	97.13	2371.7	165.3	107.74
78	2452.6	162.0	98.29	2368.5	159.0	109.82
79	2482.6	167.0	113.78	2370.5	167.8	114.42
80	2460.5	132.0	101.75	2375.0	137.0	115.38
81	2456.4	166.7	94.14	2372.3	162.2	109.56
82	2481.1	217.9	116.89	2367.7	206.8	114.51
83	2486.0	231.5	109.51	2371.9	202.7	105.85
84	2492.1	201.4	107.87	2373.7	197.8	106.70
85	2463.8	87.6	113.25	2370.5	85.1	107.31
86	2462.6	157.5	96.19	2373.8	138.0	109.53
87	2464.3	147.7	108.17	2374.3	124.7	103.68
88	2458.7	152.7	96.62	2369.9	150.7	107.53
89	2439.1	115.6	95.20	2375.4	121.1	107.16
90	2487.1	169.1	106.30	2371.7	145.1	104.19
91	2484.7	190.4	110.43	2371.8	182.2	107.79
92	2457.8	130.9	101.15	2372.4	145.1	111.15
93	2464.1	155.4	95.85	2373.4	144.4	109.09
94	2489.3	158.7	108.43	2372.7	147.1	106.15
95	2459.5	253.3	92.06	2372.9	248.2	104.23
96	2481.0	162.2	107.20	2370.1	171.9	105.43
97	2454.1	156.8	99.03	2370.7	160.7	111.53
98	2460.7	128.4	90.50	2375.0	139.3	101.96
99	2459.8	169.3	91.53	2371.8	180.6	101.89
100	2484.1	215.7	111.67	2371.7	200.5	108.46
